# ICTV Virus Taxonomy Profile: *Nanoviridae*

**DOI:** 10.1099/jgv.0.001544

**Published:** 2021-01-12

**Authors:** John E. Thomas, Bruno Gronenborn, Robert M. Harding, Bikash Mandal, Ioana Grigoras, John W. Randles, Yoshitaka Sano, Tania Timchenko, H. Josef Vetten, Hsin-Hung Yeh, Heiko Ziebell, ICTV Report Consortium

**Affiliations:** ^1^​ QAAFI, The University of Queensland, GPO Box 267, Brisbane, Queensland 4001, Australia; ^2^​ CNRS, Université Paris-Sud, CEA, 91190 Gif-sur-Yvette, France; ^3^​ Queensland University of Technology, GPO Box 2434, Brisbane, Queensland 4001, Australia; ^4^​ Division of Plant Pathology, Indian Agricultural Research Institute, New Delhi 110012, India; ^5^​ Université d'Evry Val d'Essonne, 91030 Evry, Ile-de-France, France; ^6^​ The University of Adelaide, Waite Campus, PMB Glen Osmond, SA 5064, Australia; ^7^​ Niigata University, 2-8050 Ikarashi, Niigata, 950-2181, Japan; ^8^​ Im Spargelfeld 1, 38162 Cremlingen, Germany; ^9^​ Taipei Agricultural Biotechnology Research Center, Academia Sinica, Taipei, 115, Taiwan, ROC; ^10^​ Julius Kühn-Institut, Messeweg 11-12, 38104 Braunschweig, Germany

**Keywords:** Nanoviridae, ICTV Report, taxonomy

## Abstract

*Nanoviridae* is a family of plant viruses (nanovirids) whose members have small isometric virions and multipartite, circular, single-stranded (css) DNA genomes. Each of the six (genus *Babuvirus*) or eight (genus *Nanovirus*) genomic DNAs is 0.9–1.1 kb and is separately encapsidated. Many isolates are associated with satellite-like cssDNAs (alphasatellites) of 1.0–1.1 kb. Hosts are eudicots, predominantly legumes (genus *Nanovirus*), and monocotyledons, predominantly in the order Zingiberales (genus *Babuvirus*). Nanovirids require a virus-encoded helper factor for transmission by aphids in a circulative, non-propagative manner. This is a summary of the ICTV Report on the family *Nanoviridae*, which is available at ictv.global/report/nanoviridae.

## Virion

**Fig. 1.**
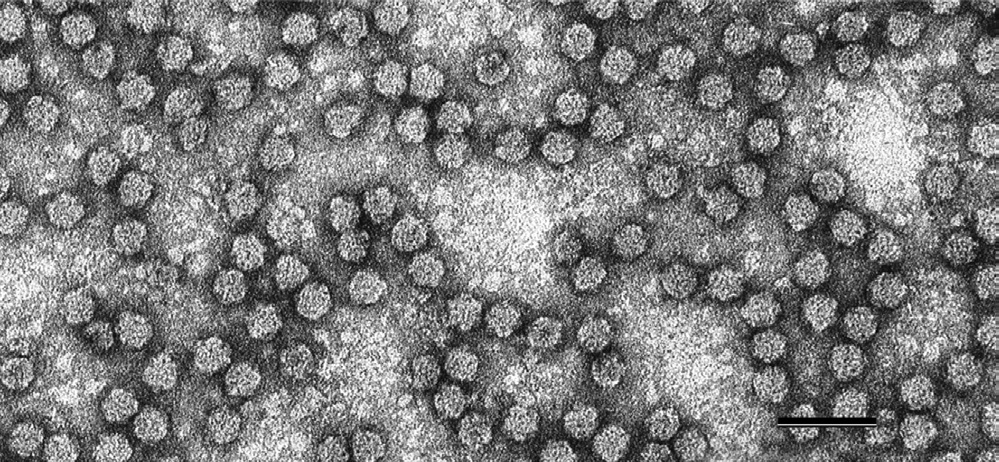
Negative-contrast electron micrograph of particles of faba bean necrotic yellows virus. Bar, 50 nm. (Courtesy of L. Katul and D.-E. Lesemann.)

Nanovirid virions are small isometric particles 17–19 nm in diameter, with a probable *T*=1 symmetry and often displaying a hexagonal profile (Table 1, Fig. 1) comprising DNA and a single species of capsid protein.

**Table 1.** Characteristics of members of the family *Nanoviridae*

Example:	subterranean clover stunt virus [AU;2534B] (MK035728–MK035735), species *Subterranean clover stunt virus*, genus *Nanovirus*
Virion	17–19 nm isometric particles, containing a single capsid protein
Genome	Multipartite, cssDNA, comprising six (each 1.0–1.1 kb; *Babuvirus*) or eight (each 0.9–1.0 kb; *Nanovirus*) components
Replication	Nuclear, by rolling-circle replication using host DNA polymerase
Translation	From transcripts of dsDNA intermediates, with the aid of host DNA and RNA polymerases
Host range	Eudicots, mainly Fabaceae (genus *Nanovirus*); monocotyledons, order Zingiberales (genus *Babuvirus*); transmitted by specific aphid vectors
Taxonomy	Realm *Monodnaviria*, kingdom *Shotokuvirae*, phylum *Cressdnaviricota*, class *Arfiviricetes*, order *Mulpavirales*; two genera including >10 species

## Genome

**Fig. 2.**
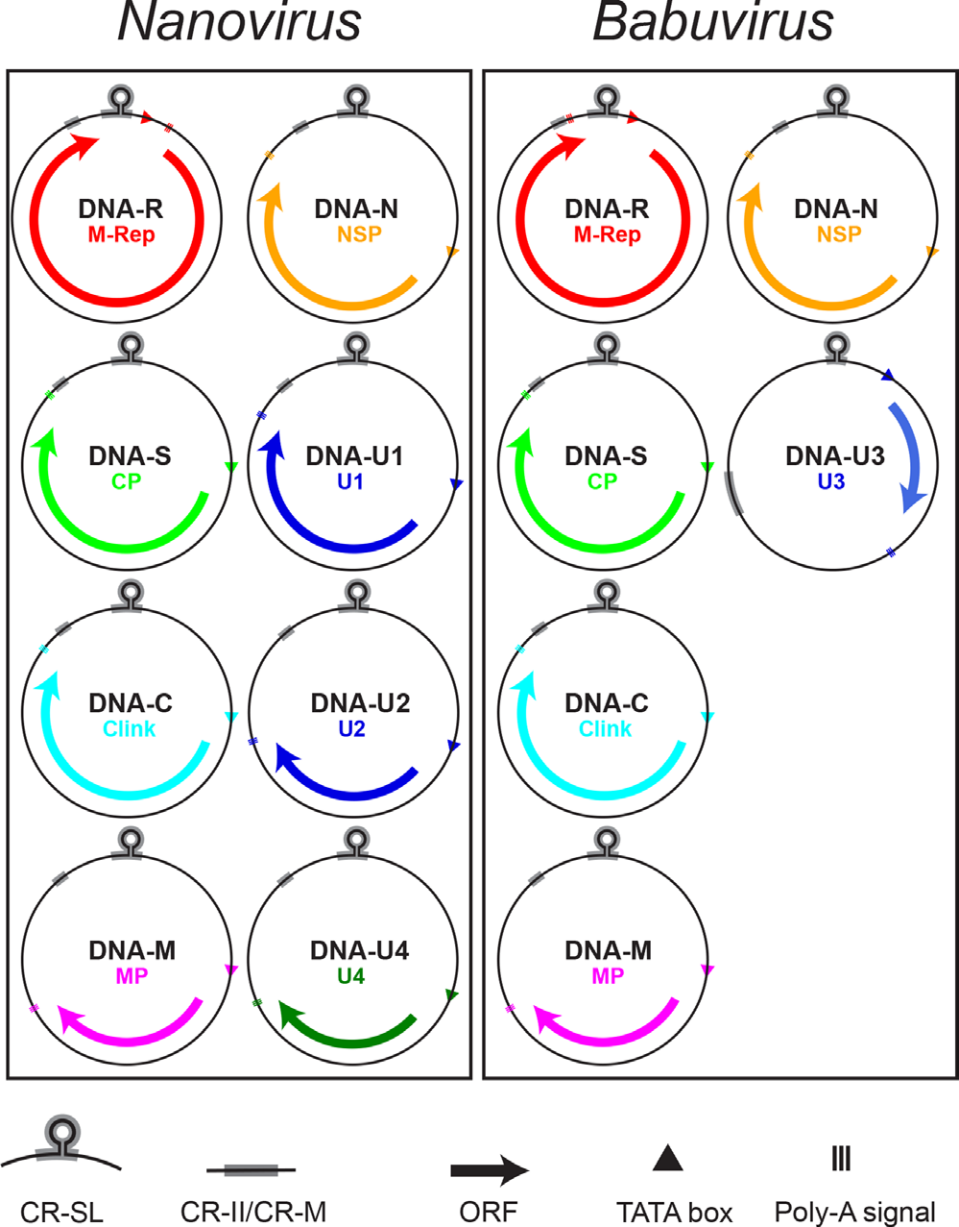
Genomic organization of viruses of the family *Nanoviridae*. DNA circles (0.9–1.1 kb) are labelled with their designated name and that of the encoded protein, with ORFs inidcated by arrows. The positions of the common stem-loop region (CR-SL) and the second common region (CR-II/CR-M) are indicated.

Based on infectivity studies, the genomes of members of the genus *Nanovirus* are thought to comprise eight independently encapsidated circular, single-stranded (css)DNA components, each of 0.9**–**1.0 kb [1]. Babuviruses have six such components, each of 1.0**–**1.1 kb. Viruses in the two genera share a set of five homologous DNA components, referred to as DNA-R (encoding M-Rep), DNA-S (capsid protein), DNA-C (Clink), DNA-M (movement protein) and DNA-N (nuclear shuttle protein) (Fig. 2). DNAs encoding proteins of unknown function have been identified from nanoviruses (DNA-U1, DNA-U2 and DNA-U4) and babuviruses (DNA-U3, potentially expressed in some banana bunchy top virus isolates only) [2]. Autonomously replicating, independently encapsidated alphasatellite molecules of 1.0**–**1.1 kb are associated with many isolates of viruses in both genera [3]. Nanovirids are unique among circulatively and non-propagatively transmitted plant viruses in requiring a virus-encoded helper factor for vector transmission by aphids [4].

## Replication

All nanovirid genomic DNAs have a similar structural organization, containing conserved inverted repeat sequences potentially forming a stem-loop structure within a common region-stem loop (CR-SL) that also contains three short repeated sequences (iterons), presumed to be binding sites for M-Rep. A second common region, conserved within virus genomes, is named CR-M (babuviruses) or CR-II (nanoviruses) (Fig. 2). Each DNA component encodes a single protein (with the single exception of banana bunchy top virus DNA-R which has a second smaller ORF, located within the larger ORF). Replication is thought to occur in the nucleus by a rolling-circle mechanism with synthesis of viral dsDNA by host DNA polymerase and mRNA transcribed by host RNA polymerase. M-Rep has DNA cleavage and nucleotidyl transferase activity and is thought to trigger replication initiation of all genomic DNAs.

## Taxonomy

Current taxonomy: ictv.global/taxonomy. Nanoviruses have mostly been isolated from legumes, while babuviruses infect monocots such as banana [5], abaca and cardamon [6]. Individual viruses are transmitted by one or a few aphid species [4].

## Resources

Full ICTV Report on the family *Nanoviridae*: ictv.global/report/nanoviridae.
